# Multi-omics insights into stem cell-mediated regeneration after traumatic spinal cord injury: cell fate, niche remodeling, and functional integration

**DOI:** 10.3389/fcell.2026.1994054

**Published:** 2026-10-29

**Authors:** Haotian Xu, Jun Wu, Chao Cheng

**Affiliations:** Department of Neurosurgery, Wuxi People’s Hospital, Wuxi, Jiangsu, China

**Keywords:** graft–host interactions, multi-omics, neural progenitor cells, neural regeneration, single-cell transcriptomics, spatial transcriptomics, stem cell transplantation, traumatic spinal cord injury

## Abstract

Stem-cell grafts may replace neural cells, protect host tissue, or change local signaling after spinal cord injury. Their effects depend on the starting cell product, how donor cells respond to the lesion, and whether they contribute to useful function. This narrative review examines neural stem/progenitor cells and neural products derived from pluripotent stem cells. Mesenchymal stromal cells (MSCs) provide a comparison for repair driven mainly by secreted factors. Transplantation studies show that regional identity and developmental stage affect graft behavior, that the lesion can redirect donor-cell fate, and that neural and stromal products can alter host responses. In selected models, neural grafts also form active connections with host circuits. Molecular profiling identifies product heterogeneity, early donor-cell states, and secreted factors associated with host responses. Injury atlases describe changes in cell populations, tissue locations, and metabolites, helping researchers choose questions for graft studies. These findings come from separate studies; no study has yet tested the full multi-omics sequence from product features to recovery. Future work should connect product measurements before transplantation to donor, host, and functional changes over time. It should test whether proposed mechanisms affect function and whether findings hold across products and recipients. Clinical translation will also require potency tests tied to intended function, long-term safety monitoring, and measures of motor, sensory, autonomic, and daily function.

## 1 Introduction

### 1.1 Regenerative challenges after traumatic spinal cord injury

Traumatic spinal cord injury (SCI) interrupts long-range neural communication and initiates persistent remodeling of the lesion and surrounding tissue. Cell replacement addresses only part of this damage: recovery also depends on where axons project and whether their connections transmit useful signals. In an experimental study of endogenous regeneration, directing axons toward appropriate targets produced a different functional outcome from growth across the lesion alone (Squair et al., 2023). The same distinction between axonal extension and effective connectivity is relevant to neural grafts.

Transplanted stem or progenitor cells may replace neural cells, support host axons, provide glial functions, or alter local signaling. Human neural progenitors have shown anatomical integration and functional effects in preclinical studies, including nonhuman primates (Rosenzweig et al., 2018). Several processes could account for these effects, including tissue preservation, axonal sprouting, remyelination, relay formation, and compensatory plasticity. Each places different demands on the graft. We therefore distinguish their contributions wherever the available evidence allows.

### 1.2 Stem cell therapy and unresolved biological questions

Cell selection begins with the intended repair mechanism. A donor-derived relay requires neurons that survive, receive host input, and transmit activity to an appropriate output. Stromal cells may instead alter host injury through a transient secretory response. The required duration of graft survival therefore depends on the role the cells are expected to perform.

Similar motor scores can arise from different mechanisms: preservation of existing projections, reconstruction of a relay, or greater recruitment of spared circuits during rehabilitation. Comparing treatments therefore requires an account of what the transplanted cells contribute within the host tissue and the functional domain being assessed.

### 1.3 Rationale, scope, and literature approach

Injury atlases show that neuronal and non-neuronal responses vary across regions and over time (Milich et al., 2021; Matson et al., 2022). As a result, an increase in a tissue transcript can have several explanations: more cells expressing that transcript, stronger expression within an existing population, or a different anatomical sampling boundary. Distinguishing these explanations is essential when a tissue-level signal is attributed to a graft.

Here, multi-omics means combining measurements from two or more molecular layers, such as RNA with chromatin accessibility or proteins. Single-cell assays show how cells differ from one another; spatial assays show where cells or molecules are located in tissue. Measuring RNA at both single-cell and spatial resolution still measures one molecular layer. Combining distinct layers may help explain why similar starting cells follow different paths after transplantation.

Recent reviews address spatial multi-omics after spinal cord injury, neural progenitor transplantation, and transcriptomic approaches to therapy development (Peng et al., 2024; Chen et al., 2026; Chudakova et al., 2026). This review asks how the starting cell product influences donor fate, host responses, and functional recovery. Section 2 describes the starting product and its repair goal. Section 3 examines how injury changes donor cells and host tissue. Section 4 explains how molecular measurements can characterize these changes, and Section 5 summarizes the mechanisms supported by current evidence. Section 6 discusses product testing and clinical outcomes. The framework allows donor–host feedback and parallel routes through tissue protection, remyelination, and neuronal relay formation. Neural products are the main focus, with MSCs providing a comparison for paracrine repair.

We used targeted web searches and reference tracing, then checked relevant records in PubMed/Europe PMC, publisher websites, and Crossref. Search terms combined spinal cord injury and stem or neural progenitor transplantation with single-cell, spatial, multi-omics, transcriptomic, epigenomic, proteomic, metabolomic, and machine-learning terms. We updated the search on September 28, 2026, without a lower date limit. Supplementary Note S1 describes the update and study-selection decisions.

We prioritized published transplantation studies that linked the cell product to donor-cell fate, host responses, or functional outcomes. Injury atlases and external methods were included only when they informed a defined graft question; selected reviews provided context. Landmark studies were retained for mechanistic or methodological contributions, while recent reports were assessed for whether they changed earlier conclusions. We included negative and contradictory findings but did not treat unpublished work or trial protocols as outcome evidence. The bibliography contains 79 records, including reviews, corrections, methods papers, and guidance documents, not 79 independent studies. The original screening denominator was not recorded and cannot be reconstructed reliably.

## 2 Initial cell products and their repair objectives

Section 2 examines the starting cell product and the repair task it is meant to perform. The regional identity of a product, developmental stage, and secreted factors help determine which donor and host changes to measure. Figure 1 pairs transplantation findings with the questions they leave open. Table 1 compares cell products by intended repair function.

**FIGURE 1:**
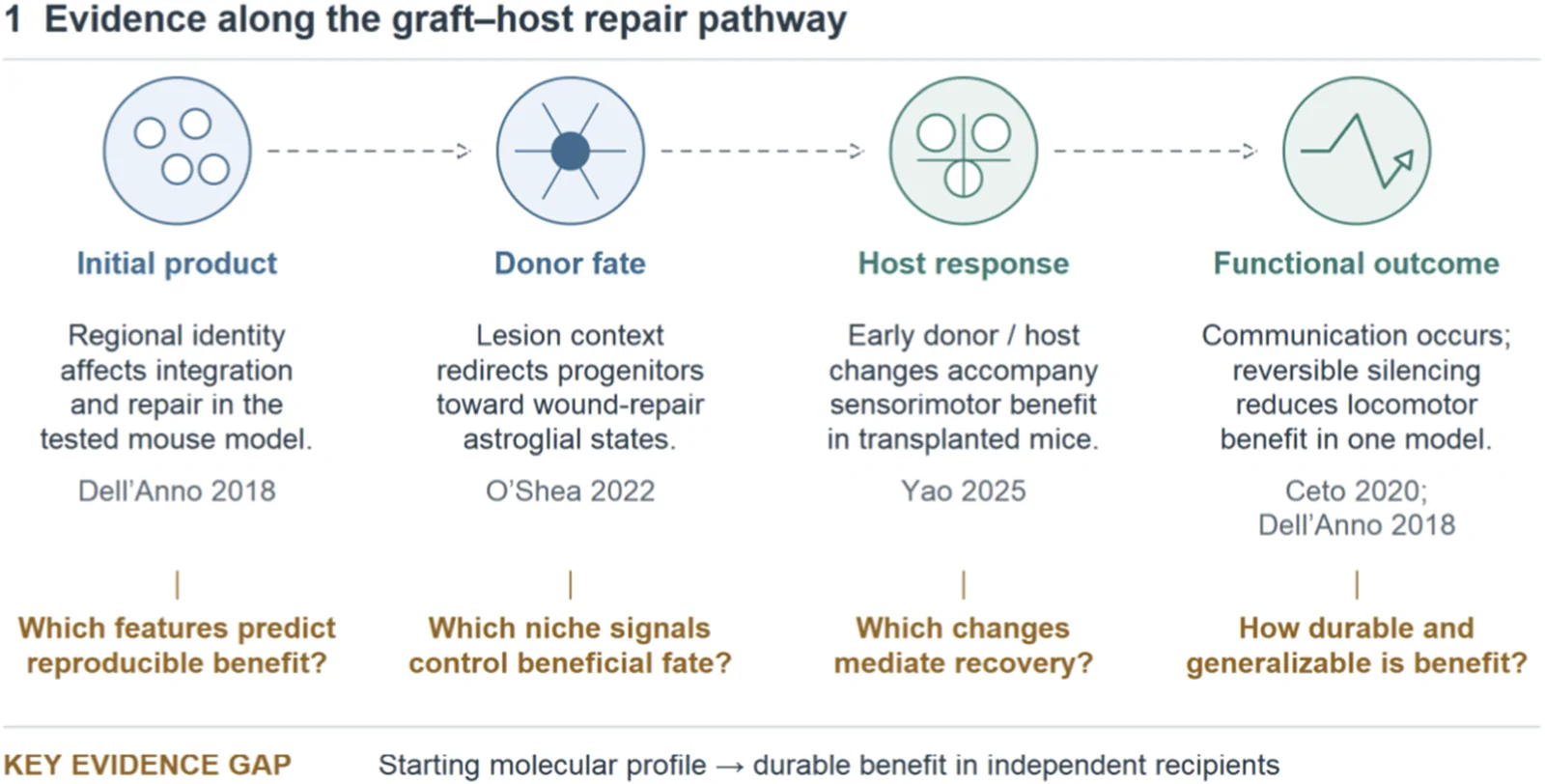
Evidence and knowledge gaps across the graft–host repair pathway.

**TABLE 1:** Cell product categories, intended repair mechanisms, and interpretation boundaries.

Product category	Principal repair objective	Relevant molecular characterization	Evidence and boundary
Tissue-derived neural stem/progenitor cells	Neuronal and glial contribution; graft–host relay	Regional identity, developmental state, neuronal–glial composition	Regional identity and developmental stage matter; anatomical growth can coexist with absent motor benefit (Dell’Anno et al., 2018; Aceves et al., 2023)
Pluripotent stem cell-derived neural products	Neural replacement, host support, and potential remyelination	Lineage restriction, residual unwanted states, maturation programs	Preclinical profiling and early clinical safety observations address different questions (Kumamaru et al., 2018; Yao et al., 2025; Sugai et al., 2026)
Mesenchymal stromal cells	Paracrine modulation and tissue protection	Secretory phenotype, source-associated variation, recipient responses	Early clinical reports do not establish donor-derived neuronal replacement (Bydon et al., 2024b)
Cell-derived secretome and extracellular vesicles (comparators)	Transfer of selected secretory activities	Protein or cargo composition and host-response measures	Cell-free products are distinct from transplantation; selected host effects do not imply complete equivalence (Romanelli et al., 2019; Vawda et al., 2020)

Categories describe biological objectives, not a ranking of clinical efficacy. Structured grafts and organoids are architectures that can contain neural products rather than a separate stem cell origin. Neural replacement, neuroprotection, and remyelination require different functional evidence.

Figure 1 places selected transplantation studies along four steps: starting product, donor fate, host response, and functional outcome. For each step, it pairs a reported finding with an open question. The categories identify evidence types, not study quality or effect size. Physiological recordings and reversible silencing test different aspects of graft function; graft dependence has been shown only in the models tested. Dashed arrows organize the review but do not show a proven causal sequence. Grafts may also affect host tissue before neural integration, and host responses may change donor fate. A key question is whether pre-transplant measurements predict lasting benefit across products and recipients. Current profiling describes product differences and donor and host changes. Cited studies appear in Sections 2, 4, and 5.

### 2.1 Neural stem/progenitor cells and pluripotent stem cell-derived products

Regional identity affects what graft-derived neural cells can contribute. Spinal and forebrain neuroepithelial grafts differed in engraftment and repair. Reversibly suppressing activity in the spinal graft reduced locomotor performance, supporting a relay contribution in that model (Dell’Anno et al., 2018). A separate study linked spinal specification of human pluripotent stem-cell-derived neural stem cells with graft integration (Kumamaru et al., 2018). These findings support regional identity as a product feature to assess. The best regional match for clinical lesions remains unknown.

The evidence needed to assess a cell product depends on its intended cell type and function. To test a neuronal relay, researchers must show that grafted neurons receive signals from the host and transmit them to an appropriate circuit. To test an oligodendroglial product, they must show that donor cells myelinate surviving axons and improve signal conduction. Because the same behavioral score can result from different repair processes, molecular tests should match the function expected of each product.

The neural products discussed here include tissue-derived populations and neural derivatives of embryonic stem cells or induced pluripotent stem cells (iPSCs). For these derivatives, pluripotency describes the source cells. The preparation actually administered must be assessed by its neural composition and developmental capacity. Table 1 groups products by repair objective to make this distinction explicit.

Earlier graft studies support the importance of cell identity and repair mechanism. Caudalized neural grafts enabled corticospinal axon growth and functional synaptic connections in rodent injury models (Kadoya et al., 2016). Human iPSC-derived neurospheres also produced neural descendants and improved motor outcomes in injured immunodeficient mice, alongside reported changes in trophic support and lesion structure (Nori et al., 2011). These findings support several possible repair processes but do not show that neuronal replacement alone caused recovery.

### 2.2 Mesenchymal stromal cells and paracrine repair

Mesenchymal stromal cell (MSC) interventions are generally interpreted in terms of host modulation rather than durable neuronal replacement. The phase I CELLTOP study evaluated an autologous adipose-derived product in ten participants, primarily to assess safety (Bydon et al., 2024b). Its exploratory neurological findings did not establish a donor-derived neuronal mechanism. Clinical outcomes from stromal and neural progenitor products should therefore be interpreted in light of their different proposed mechanisms.

In a rat contusion model, MSC-derived extracellular vesicles reproduced selected anti-inflammatory and anti-scarring changes in host tissue (Romanelli et al., 2019). This suggests that secreted MSC products can reproduce some effects of MSCs on host tissue, although vesicles and living MSCs remain distinct interventions.

Protein analysis of conditioned media compared secreted proteins from umbilical cord matrix cells, bone marrow stromal cells, and fibroblasts. The umbilical cord cell medium produced the strongest effects on selected early blood-vessel and lesion measures after cervical injury (Vawda et al., 2020). This study directly compares secretome composition with early host responses. It does not test neural replacement.

Sixteen people with chronic cervical SCI, all classified as grade B on the American Spinal Injury Association (ASIA) Impairment Scale (AIS), received autologous MSCs. In this study, reported as phase III, two participants showed segmental motor improvement over six months (Oh et al., 2016). Although this cohort cannot establish an effect across MSC products, it tempers conclusions drawn from favorable case series. Whether molecular differences between preparations explain this clinical variability remains untested.

### 2.3 Cellular heterogeneity, maturation, and product consistency

Donor background, differentiation, passage number, cryopreservation, and cell composition can change a cell preparation. Characterization should identify supportive cell types and unwanted off-target or proliferative populations. It should also test whether product composition predicts the intended biological activity across preparations. Identity, purity, and potency answer different questions; a uniform cell mixture is not necessarily the goal.

Developmental stage provides a concrete example. Aceves and colleagues compared spinal neural progenitor preparations from different stages and found differences in neuronal composition and host axon growth. Later-stage grafts worsened thermal hypersensitivity, while transplantation did not significantly improve locomotor recovery relative to the comparison groups in that model (Aceves et al., 2023). The anatomical changes did not amount to an overall functional benefit. The sensory harm also illustrates why studies aimed at motor repair need to assess more than locomotion.

Human neural grafts matured over prolonged periods in primates (Rosenzweig et al., 2018). Product comparisons therefore need follow-up long enough to distinguish persistent developmental differences from temporary delays. Linking early profiles to later graft composition is more informative than comparing products at a single endpoint.

A later primate study reported better skilled forelimb recovery in animals with surviving human spinal neural stem-cell grafts (Sinopoulou et al., 2026). Because this comparison involved animals with and without surviving grafts rather than randomized treatment groups, it cannot estimate a general treatment effect. The findings suggest that durable graft survival may matter when relating product features to recovery. More studies are needed to determine how consistently this relationship holds.

Outside SCI, droplet-based RNA profiling measured asynchronous differentiation in embryonic stem cells (Klein et al., 2015). This method could identify cells at different developmental stages before transplantation. Together, the graft studies reviewed above show that cell identity and developmental stage can affect repair. The best combination likely depends on the intended repair function.

## 3 How the host niche shapes donor fate and tissue responses

### 3.1 Neuroinflammation and immune–glial responses

When a cell product enters an injured spinal cord, it encounters a changing local environment. Single-cell studies identify immune, astroglial, fibrotic, and vascular cell populations after injury (Milich et al., 2021). Their locations and changes over time provide a baseline for examining donor survival, maturation, and host responses after transplantation.

Scar-forming astrocytes can support axon growth under growth-stimulating conditions. Microglia at the lesion border help organize the neuroprotective scar that preserves surrounding tissue (Anderson et al., 2016; Bellver-Landete et al., 2019). Broadly suppressing these responses could remove tissue-protective functions. Graft studies should distinguish host responses that support repair from those that prolong injury.

For graft studies, distinguish an increase in the number of host cells from a change in what those cells do. The same cell populations may remove debris, contain the wound, or contribute to ongoing injury. Their location and measured function therefore provide more useful information than a broad label such as inflammation.

Donor death, maturation, and secretion can change which host cells are recruited. In turn, host responses can affect which donor cells survive. Measuring donor and host cells together over time helps reveal this two-way relationship. An untransplanted injury atlas provides the reference; comparison with transplanted tissue identifies changes associated with the product.

Microglial depletion studies show that changing host microglial states can alter recovery (Hakim et al., 2021). Intervention studies identify interactions between immune and glial cells (Brennan et al., 2022). Peripheral macrophages can also limit microglial activation (Greenhalgh et al., 2018). These injury studies suggest which host interactions to examine during transplantation. Graft experiments still need to show whether preserving or changing these populations affects the product’s benefits.

Studies of scar-free neonatal repair and adult microglial transplantation describe coordinated matrix formation and resolution of inflammation (Li et al., 2020). In a more direct transplantation study, neural precursor grafts changed phagocyte responses and reduced secondary damage after mouse contusion (Cusimano et al., 2012). These findings support measuring host immune responses alongside donor-cell lineages, because grafts may protect tissue before or while neural replacement occurs.

### 3.2 Extracellular matrix, scarring, and vascular support

The Tabulae Paralytica combines single-nucleus RNA sequencing, paired RNA–chromatin profiling, and spatial measurements. In old mice, the study also tested gene therapy that restored the blood–spinal cord barrier and natural walking recovery (Skinnider et al., 2024). For graft studies, it points to wound containment as a host feature to measure and preserve when modifying the lesion. The gene-therapy experiment tested host repair, not a transplantation strategy.

Experiments that separate intrinsic growth capacity, the supporting substrate, and guidance show that axon regeneration depends on all three (Anderson et al., 2018). For graft studies, this means testing whether the lesion matrix provides routes for host axons to enter and donor axons to extend. Total matrix abundance alone cannot show whether those routes support growth.

In rats, MSC-derived extracellular vesicles entered macrophages and were associated with altered transforming growth factor beta signaling and reduced blood–spinal cord barrier permeability (Nakazaki et al., 2021). This links the secreted product to macrophage and vascular responses. It does not show that new vessel growth caused recovery or that neural grafts produce the same response.

Endogenous studies show that pericyte-derived cells help seal the lesion (Göritz et al., 2011). Reducing fibrosis from these cells improved axonal and functional outcomes in another study (Dias et al., 2018). A third study found that STAT3-dependent astrocytic borders contain inflammatory and fibrotic cells (Wanner et al., 2013). Together, these findings suggest a specific graft strategy: preserve wound containment while testing whether selected barriers prevent donor or host axons from connecting. They do not support removing scar tissue indiscriminately.

### 3.3 Metabolic adaptation and bidirectional communication

Grafts need energy to survive, grow, and mature, but injury can disrupt blood flow. RNA measurements show which metabolic genes cells express; metabolite assays show which molecules are present in tissue. Neither measurement alone shows how quickly a pathway operates or which cell produced a tissue metabolite. Donor cells, immune cells, and surviving neural cells all share the lesion environment.

Spatial maps of RNA and metabolites identify lesion regions where researchers can test donor survival and maturation (Dong et al., 2025). A transplantation study could compare donor persistence across these regions and measure whether grafting changes local metabolite levels. Identifying the producing cell type and measuring pathway activity would require additional experiments.

Together, transplantation studies show two-way communication: injury changes donor-cell behavior, and grafts can change host responses. Current graft evidence most clearly supports immune changes and tissue protection. Whether altered metabolism helps graft cells survive remains unknown. This distinction guides the choice of assays in the next section.

## 4 Molecular measurements that connect product state to graft and host behavior

We group assays by the question they answer. RNA and chromatin assays describe product composition and donor-cell state. Protein and metabolite assays measure candidate effectors and local tissue conditions. Spatial assays show where these cells and molecules are located relative to the graft. Figure 2 links selected measurements to reported functional findings and identifies graft experiments that are still needed. Table 2 separates direct graft studies from injury atlases and secretome comparisons.

**FIGURE 2:**
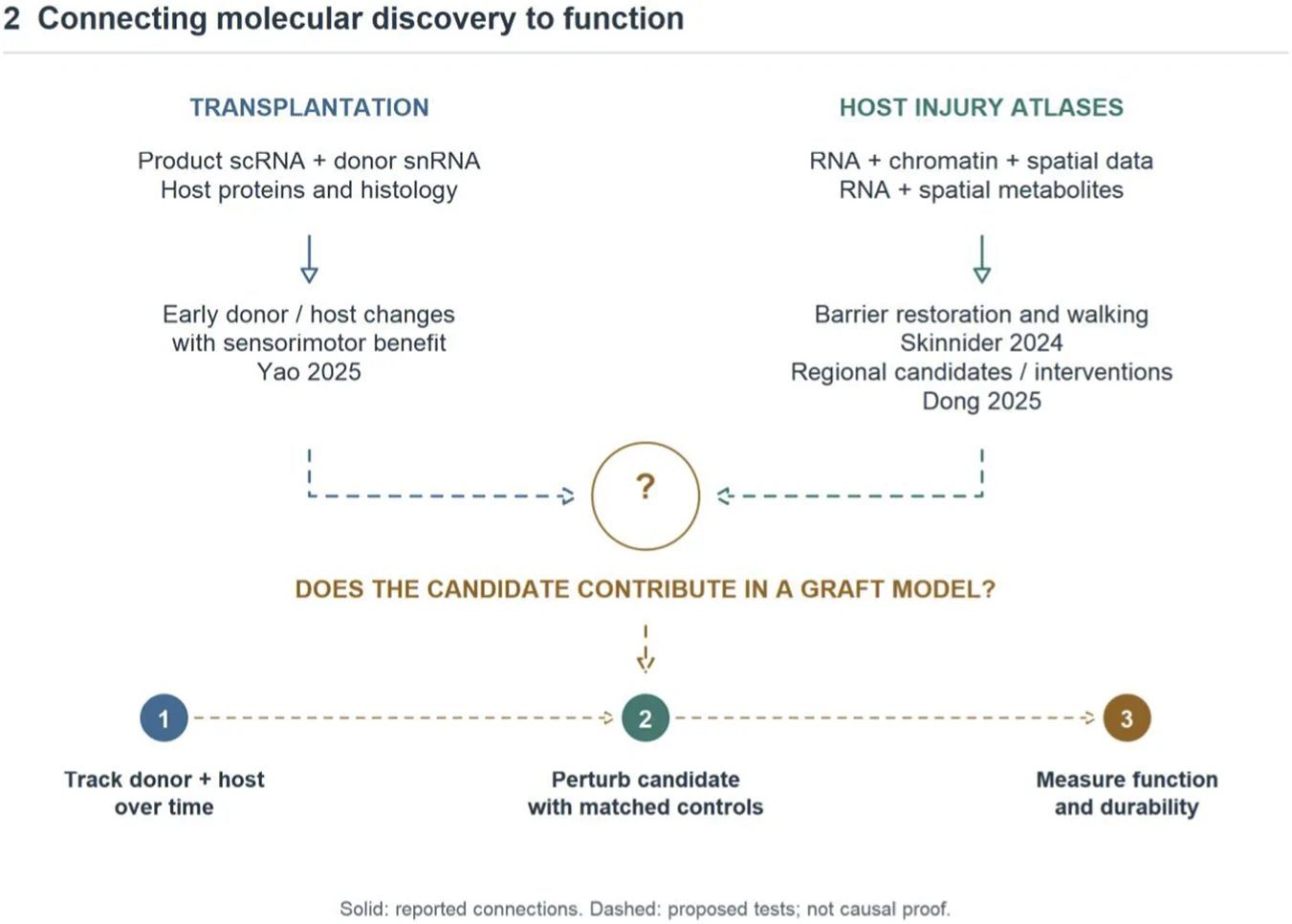
Connecting omics measurements to functional evidence and graft-specific tests.

**TABLE 2:** Selected primary molecular studies relevant to spinal cord injury and cell-mediated repair.

Study	Setting and evidence scope	Molecular or functional approach	Contribution and main limitation
Milich et al. (2021)	Mouse injury; endogenous host response	Single-cell RNA profiling and interaction inference	Resolves lesion heterogeneity; inferred communication is not equivalent to demonstrated signaling
Skinnider et al. (2024)	Mouse injury; multimodal atlas and intervention studies	Single-nucleus RNA, paired RNA–chromatin, spatial atlases	Adds regulatory and spatial context to host states, with barrier-restoration experiments; graft effects remain untested
Dong et al. (2025)	Rat injury atlas with additional mouse and *in vitro* validation	Single-cell RNA in separate samples; spatial RNA and metabolites in adjacent sections	Adds biochemical context; selected copalic acid and IGF2 effects were tested. Regional enrichment does not establish cellular source or graft response
Yao et al. (2025)	Human neural progenitors in immunodeficient mice	Pre-transplant single-cell RNA; donor-nucleus RNA at 1 week post-transplant; host protein/histology and functional assessments	Profiles early donor RNA states alongside host protein and histological responses; does not jointly map donor and host transcriptomes or isolate the cause of functional improvement
O’Shea et al. (2022)	Mouse NPCs transplanted into CNS lesions, including subacute SCI	Donor-selective RiboTag RNA profiling; comparison with uninjured tissue	Links lesion context to a wound-repair astroglial phenotype; does not establish motor benefit or a universal graft fate
Vawda et al. (2020)	Rat cervical injury; cell-conditioned media comparison	Cytokine/chemokine/angiogenic protein profiling plus acute tissue measures	Connects secretory composition with selected vascular outcomes; not a paired multi-omics transplantation study

Evidence is classified by the layers actually measured. RNA and spatial RNA provide complementary resolution within one molecular layer; transcriptomics plus chromatin or metabolomics adds a distinct layer. Endogenous injury atlases do not establish graft mechanisms. Yao et al. published a correction affecting quantitative panels 5B and 6B and group identifiers in panel 7B (Yao et al., 2026); interpretation of those panels requires the corrected version. Study-specific biological sample sizes differ by experiment and endpoint and are not pooled in this table. Milich et al. published a correction to the panel labels in Figure 5 of their original article and the APOE high/low legend; the correction states that the conclusions are unchanged (Milich et al., 2025).

Yao and colleagues linked pre-transplant product and early donor RNA profiles to host protein and tissue outcomes (Yao et al., 2025). In old mice, Skinnider and colleagues paired a host-injury atlas with barrier-restoring gene therapy and recovery of walking (Skinnider et al., 2024). Dong and colleagues mapped RNA and metabolites across injured tissue to identify candidate regions and pathways (Dong et al., 2025). These studies raise different questions for transplantation. The latter two do not demonstrate neural graft mechanisms. Arrows connect measurements to reported findings, not necessarily causal effects. Spatial RNA remains an RNA assay, and separate samples or adjacent sections do not capture the same cells. The lower sequence proposes longitudinal measurements and functional tests; it is not a validated predictor. See Table 2 for the correction to the Yao study.

### 4.1 Single-cell transcriptomics and epigenomics

Yao and colleagues studied human iPSC-derived spinal neural progenitors before transplantation, then profiled donor nuclei one week after grafting into injured immunodeficient mice (Yao et al., 2025). They also measured host proteins and tissue changes. This study compares the starting product with donor states at one early time point. Longer follow-up and studies in an intact immune system are needed to assess later maturation and recipient responses.

Several technical problems can distort molecular profiles from grafts. Free RNA in a sample, called ambient RNA, can be mistaken for RNA from a cell. A droplet may also contain more than one cell. In addition, some cell types may be recovered less efficiently than others. Species-aware sequence assignment and histology-based cell counts can help check whether donor lineages or host populations truly changed, especially when a rare state appears or disappears.

Paired RNA and chromatin-accessibility measurements show whether gene-expression changes occur alongside changes in chromatin, as in the Tabulae Paralytica (Skinnider et al., 2024). In grafts, these measurements could show whether early RNA changes persist and are accompanied by chromatin changes. Perturbing a candidate regulator would then test whether it contributes to donor-cell fate.

Trajectory and RNA-velocity methods, including scVelo, infer possible transitions between cell states from RNA data (Bergen et al., 2020). For donor-cell maturation, these predictions can guide which time points to sample. Researchers should compare them with lineage tracing and physiological development. An inferred sequence does not track individual cells over time.

The number of independent donors or recipients matters more for generalizing an association across products than the number of cells measured from one preparation. Bulk assays compare preparations or recipients; single-cell assays show variation within each sample. Profiling more cells improves resolution within a sample, but does not add independent transplantation outcomes.

SHARE-seq provides an example of measuring RNA and chromatin accessibility in the same cells (Ma et al., 2020). This approach could compare product and graft-cell states. Its original lineage predictions do not establish what happens to cells transplanted into the spinal cord.

### 4.2 Proteomics, secretomics, and metabolomics

Protein assays can test whether RNA changes are accompanied by changes in proteins that cells make or release. Joint RNA and selected cell-surface protein measurements can refine cell identity (Hao et al., 2021); broader proteomics measures secreted and intracellular proteins beyond a selected antibody panel. For graft studies, choose the assay based on whether the question concerns donor-cell function or a response in host tissue.

For a product expected to act through secreted factors, measure its secretome alongside responses in recipient cells. Interpretation also depends on which cells produced the factors, whether they reach the lesion, and how many viable donor cells remain. A larger total amount may simply reflect better donor survival. Reproducible changes in recipient-cell activity linked to secretome composition are therefore more informative than measuring one candidate factor alone.

Dong and colleagues combined single-cell RNA sequencing with spatial RNA and metabolite maps to identify injury-associated cell populations and biochemical regions (Dong et al., 2025). They used separate rat samples for single-cell assays and adjacent tissue sections for spatial measurements. Additional mouse and cell-culture experiments tested copalic acid-associated inflammatory responses and IGF2-associated protection and recovery. These findings nominate lesion conditions to test around grafts; they do not yet show how a transplanted product changes local metabolism or donor persistence.

Uridine was identified through cross-species metabolomics and tested in non-SCI tissue-regeneration models (Liu et al., 2022). Testing whether uridine supports donor-cell survival after spinal cord injury is a separate, untested question.

Cellular indexing of transcriptomes and epitopes by sequencing (CITE-seq) provides another transferable tool: joint measurement of transcripts and selected surface epitopes (Stoeckius et al., 2017). It could help verify donor and host phenotypes, but its targeted panel cannot substitute for secretome or broad proteomic analysis.

### 4.3 Spatial multi-omics and regenerative niches

Spatial measurements locate donor and host cells relative to lesion borders, blood vessels, and surviving axons. Combining these maps with single-cell references can identify candidate populations and variation within each population (Kleshchevnikov et al., 2022; Lopez et al., 2022). For transplantation, the key question becomes which cells changed and whether their location could affect graft survival or integration. The answer is limited by the resolution of the assay and the quality of the reference atlas.

Adult reference atlases may not include immature cell states found in a graft or states created by injury. Assigning every profile to a known cell type can hide these unfamiliar states. Researchers should inspect unmatched profiles and confirm them with an independent method. Adjacent sections can be aligned by anatomy, but they do not contain the same individual cells.

Ligand–receptor and ligand–target analyses use known molecular interactions to nominate signals that donor cells may send to host cells, and *vice versa* (Browaeys et al., 2020; Jin et al., 2021). These analyses produce candidate lists; they do not measure every signal that is active. Researchers can use the lists to select interactions for testing at the graft interface.

To test a candidate interaction, researchers can check whether donor and host cells are near each other and whether the host cell has the proposed receptor. They can then measure the host-cell response and test whether disrupting the proposed signal changes that response. These steps help distinguish a functional signal from a predicted interaction.

The original spatial-transcriptomics method measures RNA while preserving its location in tissue (Ståhl et al., 2016). Its value for graft studies is to show where expression changes occur. To call an analysis spatial multi-omics, researchers must add another molecular layer and specify whether measurements correspond across cells, sections, or samples.

### 4.4 Artificial intelligence and reproducible multi-omics integration

Artificial intelligence (AI) methods can compare product, donor, and host measurements when some samples lack one or more molecular assays. MultiVI integrates paired and unpaired molecular data (Ashuach et al., 2023). Its original validation did not include spinal cord transplantation. These methods can estimate values for measurements that are missing. Analyses should label observed and estimated values separately and carry uncertainty into later experiments.

Animals or donors, rather than individual cells, should define independent replication (Squair et al., 2021). Integration must also balance batch removal against retention of biological differences (Luecken et al., 2022). In graft studies, manufacturing batch, donor background, injury stage, and sequencing run can be confounded. Products and treatment groups should therefore be distributed across processing batches; correction cannot reliably recover a treatment effect when treatment and batch are inseparable.

When comparing platforms, report which samples were compared, the genome and feature identifiers, the quality filters and normalization steps, and which measurements were missing. Paired measurements provide stronger links than adjacent sections or unmatched groups. Check the integrated data against known donor identity and injury stage, and against independently measured protein or spatial features, to ensure integration has not erased real product differences.

For reproducible analyses, report raw-data accessions, sample metadata, code, software versions, model settings, and random seeds. When donor or animal numbers are small, hold out entire biological samples during machine-learning evaluation; perform feature selection and tuning only within the training data. Testing on a separate donor or recipient is more informative than splitting cells from the same animal across training and test sets. Table 3 compares platform capabilities, limitations, and the graft question each can address.

**TABLE 3:** Omics platforms and their uses in SCI transplantation.

Platform	Main contribution	Principal limitation	Use in SCI transplantation
Single-cell or single-nucleus RNA sequencing	Resolves donor and host populations and transcriptional states	Dissociation or nuclear sampling bias; RNA abundance is not activity; rare cells may be missed	Compare starting product with recovered donor states; distinguish host recruitment from state changes
Paired RNA and chromatin accessibility	Links expression to candidate regulatory programs in matched cells	Sparse coverage, assay cost, and inferred regulatory links	Test stability of neural identity and nominate fate regulators for perturbation
Spatial transcriptomics	Places donor and host states within lesion architecture	Resolution, capture efficiency, segmentation and reference dependence vary	Locate graft borders, vascular niches and candidate interactions; validate donor origin
Proteomics and secretome profiling	Measures proteins and candidate extracellular effectors	Dynamic range and mixed sources; abundance does not establish release, localization or activity	Compare secretory products and test receptor engagement or functional recipient responses
Metabolomics and spatial metabolomics	Measures biochemical environments and spatial metabolite patterns	Handling sensitivity, incomplete annotation and limited source attribution; concentration is not flux	Identify graft niche constraints, then test uptake, pathway activity and donor survival
RNA with antibody-based protein measurements	Links transcriptional state with selected protein markers	Restricted antibody panels, specificity and epitope accessibility	Refine product identity or host phenotyping; verify assay transfer across batches

This platform comparison summarizes methodological capabilities discussed in Section 4, not comparative efficacy. Spatial RNA and single-cell RNA measure one molecular layer; pairing RNA with chromatin, protein, or metabolites adds a distinct layer. Platform choice should follow the product–donor–host–outcome question.

## 5 What current evidence supports along the repair pathway

Transplantation studies support distinct steps in repair, but not every link. Donor-specific profiling shows how graft cells change. Tissue and lineage studies support host-tissue protection and remyelination. Physiological recordings test whether grafts communicate with host circuits. Reversible silencing and graft removal test whether graft activity or graft presence contributes to function. The following sections distinguish these findings from mechanisms that remain untested.

### 5.1 Graft survival, lineage commitment, and neuronal maturation

Studies show that spinal identity affects neural graft behavior (Dell’Anno et al., 2018; Kumamaru et al., 2018). A key question is whether donor cells retain or change that identity after transplantation. Comparing the starting product with donor RNA and chromatin profiles over time could answer this question.

Researchers can assess graft maturation by asking whether molecular markers and cell function agree. RNA and chromatin profiles describe developmental state; protein measurements and physiological tests show whether donor cells acquire the properties needed for their intended role.

Absolute donor-cell counts and lineage tracing help distinguish cell differentiation from selective survival. They also connect the cell profiles at the study endpoint to the product that was administered.

Donor-specific RiboTag profiling, which captures ribosome-associated RNA from graft-derived cells, showed that neural progenitors acquired a wound-repair astroglial phenotype in mouse lesions, including subacute SCI (O’Shea et al., 2022). This shows that the injured host can redirect donor-cell fate. It does not establish that every neural graft becomes astroglial or that this change improves motor function.

Long-term graft studies show that human neural cells can continue to mature over time. Human neural stem cells continued to mature in injured immunodeficient rats, and functional improvement appeared late in follow-up (Lu et al., 2017). A separate study reported long projections and anatomical contacts between human iPSC-derived neural cells and host neurons (Lu et al., 2014). Together, these findings show that extensive growth and anatomical contact do not by themselves establish mature circuit function.

### 5.2 Neuroprotection, niche remodeling, and remyelination

Studies of human spinal neural progenitors report donor differentiation, changes in host tissue, and sensorimotor outcomes in immunodeficient mice (Yao et al., 2025). These results support contributions from both donor and host responses. Selective experiments are needed to measure their separate contributions; the shared outcome alone cannot be attributed to neuronal replacement.

Tissue preservation retains host axons that can support conduction and later plasticity. Structural preservation and donor-lineage measurements therefore help distinguish this route from the formation of a new relay.

To test remyelination, studies should follow four steps: confirm that axons survive, identify which donor or host cells make myelin, examine the resulting myelin, and test whether conduction improves. Myelin-related RNA can identify candidate cells, but cannot establish this sequence on its own.

Human embryonic stem cell-derived oligodendrocyte progenitors promoted remyelination and motor recovery when transplanted early after rat SCI. They did not show the same benefit in a late chronic model (Keirstead et al., 2005). Human neural progenitors selected for oligodendrocyte differentiation also promoted tissue sparing and remyelination in injured rats (Nagoshi et al., 2018). Both studies reported tissue protection and myelin repair, so they cannot establish how much each process contributed to recovery.

Resident oligodendrocyte precursor cells produce substantial new myelin after contusion (Assinck et al., 2017). In a separate mouse study, inhibiting much of this remyelination did not prevent spontaneous stepping recovery (Duncan et al., 2018). Transplantation studies should distinguish host myelin from myelin made by graft cells and test whether each contributes to the measured outcome. Together, these studies show graft-associated tissue sparing and remyelination in some models, but do not establish how much either process improves behavior.

### 5.3 Axonal growth, synapse formation, and functional recovery

Functional imaging and stimulation showed activity within graft networks and communication with host circuits (Ceto et al., 2020). Molecular profiling can identify the participating neurons, while projection tracing can distinguish host input, donor output, and spared host fibers.

In a mouse contusion study, grafted spinal neurons connected to hindlimb motor circuits, and chemogenetic activation triggered muscle activity in some animals. Transplantation alone did not improve locomotion (Tucker et al., 2025). A 2026 rat study reported synaptic integration and motor recovery after grafting human spinal interneurons, using RNA profiling, axon tracing, and electrophysiology (Zholudeva et al., 2026). Single-cell and single-nucleus RNA assays still represent one molecular layer. Together, these studies show that grafts can engage motor circuits, but circuit connections do not always improve behavior.

Directed endogenous regeneration showed that appropriate target engagement was required for useful recovery (Squair et al., 2023). Its specific relevance to transplantation is the choice of test: a proposed donor relay should be evaluated for output location and recruitment of the receiving circuit, not just axonal extension.

In the study described in Section 2.1, silencing spinal grafts reduced locomotor performance. The result supports an activity-dependent graft contribution in that model, but does not establish relay formation as a general mechanism across products.

Removing human neural stem-cell grafts abolished locomotor benefit in injured immunodeficient mice (Cummings et al., 2005). Thus, graft cells were required for benefit in that model, but the experiment cannot separate neuronal from glial contributions. Recordings, reversible silencing, and graft removal answer different questions: whether grafts communicate with host circuits, whether graft activity supports behavior, and whether graft cells are required. These conclusions apply only to the products, lesions, and outcomes tested.

## 6 Translating the repair pathway into product and clinical decisions

Clinical translation requires tests showing whether product features predict donor and host responses that lead to meaningful benefit. Product assays should measure the biological activity the cells are intended to provide. Studies must also account for recipient biology and long-term benefits and harms. Table 4 lists research priorities, not validated criteria for choosing products or recipients.

**TABLE 4:** Translational barriers and the evidence needed to address them.

Barrier	Potential contribution of multi-omics	Additional evidence needed	Interpretation limit
Product heterogeneity	Define regional and developmental composition	Reproducibility across donors/batches and mechanism-linked potency	Identity and purity do not independently establish potency
Variable host niches	Resolve immune, glial, vascular, and metabolic states	Product-specific comparisons at defined injury stages	A recovery-associated host state is not necessarily a treatment-benefit marker
Weak functional attribution	Connect donor/host programs to candidate mechanisms	Lineage, anatomy, physiology, and relevant behavioral evidence	Molecular change is not proof of circuit contribution
Cross-species and sampling differences	Compare conserved and divergent states	Appropriate immune context, maturation times, and tissue-to-fluid bridging	Accessible biofluids are not direct substitutes for lesion tissue
Long-term safety and clinical uncertainty	Identify candidate undesirable product states	Longitudinal surveillance and adequately controlled clinical studies	Rare adverse outcomes cannot be excluded by small phase I cohorts (Bydon et al., 2024b; Sugai et al., 2026)
Outcome mismatch	Identify mechanisms relevant to distinct domains	Motor, sensory, autonomic, pain, and daily-function assessment	Improvement in one domain can coexist with harm in another

This is a synthesis of unresolved research requirements, not a validated clinical pathway. The corrected CELLTOP record gives a mean injury-to-injection interval of 12 months (range 7–22 months) (Bydon et al., 2024a).

### 6.1 Cell product selection, quality, and potency

Discovery profiles can identify candidate product features, such as regional identity, maturation, secreted proteins, or unwanted cell types. A potency assay should test whether the product performs its intended biological function. U.S. Food and Drug Administration (FDA) guidance treats potency testing as product-specific and does not prescribe one assay or universal acceptance criterion for all cellular products (U.S. Food and Drug Administration, 2011). A transcriptomic identity score may help characterize the product, but it does not demonstrate that the product performs its intended function.

A potency marker is useful only if it predicts biological activity across independent donors and manufacturing batches. The routine assay must also give consistent results after storage and handling. If manufacturing changes, compare product features and biological activity before and after the change. Broad molecular profiles are most useful when they identify a small set of markers that still predicts activity under these conditions.

Good manufacturing practice (GMP) requires traceable materials and defined controls for each cell product. Relevant tests may assess identity, purity, viability, sterility, genetic stability, and residual pluripotent or proliferative cells. Which attributes matter depends on the product. Developers should discuss manufacturing and preclinical evidence with the relevant regulator as development progresses (International Society for Stem Cell Research, 2025). Proposed release tests could measure neural maturation or a reproducible response in recipient cells. Each assay still needs product-specific validation.

Transcriptomic findings also illustrate why safety assessment must extend beyond early benefit. One human iPSC-derived neural preparation initially improved function but subsequently formed tumors, accompanied by motor deterioration; transcriptome analysis implicated mesenchymal transition in this abnormal behavior (Nori et al., 2015). The observation was specific to the tested clone and preparation. It nevertheless demonstrates that an early neural phenotype and functional improvement cannot establish long-term product safety.

### 6.2 Transplantation timing and combination strategies

Select recipients based on lesion biology as well as time since injury. Measurements of wound, vascular, and immune states may help identify which recipients are likely to respond to a specific product. These predictions must be tested in transplantation studies.

Acute, subacute, and chronic injury describe changing biological conditions; they do not correspond to the same number of days in every species or model. Supplementary Figure S1 relates these phases to host measurements and graft questions. Studies should also report time since transplantation, because an immature graft may be placed into a chronically injured spinal cord.

Combination treatments should be selected to address a specific limitation of the graft. For example, a neuronal graft may need support for axon growth, a permissive substrate, or guidance toward appropriate host targets. Studies of endogenous axon regeneration distinguish these requirements (Anderson et al., 2018). They do not show which support a particular cell product needs.

Biomimetic scaffolds supported reconstruction involving neural progenitors in experimental SCI (Koffler et al., 2019). Engineered thoracic spinal cord organoids provide another way to organize graft cells by region and dorsal–ventral position (Zhu et al., 2026). In that study, spatial RNA measurements after transplantation described neuronal maturation and location. The grafts also showed electrophysiological activity and improved hindlimb movement in mice. These findings link graft architecture to molecular and functional observations, but do not isolate the effect of architecture from other product features.

Researchers used regenerative gene-expression signatures to identify thiorphan as a candidate treatment partner, then tested the approach in experiments that included neural grafting (van Niekerk et al., 2025). This study links transcriptomic candidate selection with transplantation and functional testing; it does not validate an integrated multi-omics pathway. The proposed combination pairs host neurons in a growth state with support from the graft. Testing other products should begin by asking whether they face the same limitation.

Studies combining stimulation and rehabilitation identify host neurons involved in recovery (Kathe et al., 2022). For graft trials, this means that rehabilitation and stimulation may affect how spared circuits contribute to outcome. Trials should standardize or record these treatments and directly test whether they improve graft outcomes.

In immunodeficient mice, human neural stem cells improved locomotion when transplanted during an early chronic contusion stage (Salazar et al., 2010). A separate study found no comparable benefit when oligodendrocyte progenitors were transplanted much later after injury (Keirstead et al., 2005). The studies used different cell types, recipients, and injury stages, so they cannot establish a single transplantation window.

### 6.3 Clinical evidence, safety, and meaningful outcomes

Clinical studies have so far focused mainly on safety and feasibility. CELLTOP followed ten recipients of autologous adipose-derived stromal cells for 96 weeks. No serious adverse events were reported, but investigators observed non-serious symptoms and cauda equina imaging changes; neurological outcomes were exploratory (Bydon et al., 2024b). In 2026, an open-label study treated four participants with subacute, complete cervical injury using iPSC-derived neural progenitors. Investigators reported no tumors or graft-related adverse events during follow-up (Sugai et al., 2026). The study also recorded two moderate surgical adverse events and 22 mild or moderate events attributed to tacrolimus. These are event counts, not participant counts.

Follow-up of the four participants continued for 2–4 years after transplantation, but investigators did not directly confirm graft survival, differentiation, or integration in the recipients (Sugai et al., 2026). Longer observation can detect delayed events, but four participants provide little information about uncommon risks. Registry comparisons may also reflect differences in prognosis or care. This study and CELLTOP do not establish a multi-omics test for recipient selection or recovery prediction.

A separate 2026 report followed three recipients of neural stem cells for chronic cervical SCI for 60 months, reporting no tumor formation and limited functional changes (Abraham et al., 2026). This adds long-term observations for a distinct cervical cohort, rather than enlarging the previously reported thoracic cohort. Its uncontrolled design and rehabilitation exposure prevent attribution of the observed changes to transplantation alone.

Product testing and recipient follow-up address different safety risks. Sampling may miss rare unwanted cells, and the host environment may change donor-cell behavior. Safety assessment should consider immune rejection and the treatment needed to control it, as well as tumors, abnormal growth, pain, and autonomic effects. Immunodeficient animal models cannot fully capture these immune risks. Follow-up should combine imaging, neurological examinations, adverse-event reports, and immunosuppression records for as long as the product persists or matures.

Clinical outcomes should measure the proposed repair effect and its consequences for patients. Motor scores alone may miss sensory loss, pain, or changes in daily activity. Combining anatomical and conduction measures with motor, sensory, autonomic, and daily-function assessments gives a fuller account of benefit and harm.

A phase I study of four participants with SCI lacked a control group and was too small to determine whether transplantation caused functional changes (Curtis et al., 2018). A later report followed the same participants for up to five years. This adds longer follow-up, not an independent cohort or a controlled test of efficacy (Martin et al., 2024). One participant died from sepsis associated with a sacral ulcer 30 months after treatment. The authors considered the graft or surgery an unlikely cause but could not exclude a contribution from immunosuppression. A multicenter study of chronic cervical injury ended early when motor outcomes did not meet the efficacy threshold set by the sponsor (Levi et al., 2019). An open-label study of oligodendrocyte progenitors in 25 participants supported further evaluation but also reported serious events related to surgery and immunosuppression (Fessler et al., 2022). Because the products and study designs differed, these results cannot be combined into one estimate of efficacy.

Patient priorities provide a further basis for outcome selection. A survey of people living with SCI identified arm and hand function, sexual function, and bladder and bowel function as important concerns, with differences between injury groups (Anderson, 2004). Although historical, the study supports assessing benefit beyond walking and total motor scores, alongside consultation with current trial participants.

## 7 Discussion and future perspectives

Figure 3 outlines experiments to test whether recovery depends on relay activity, donor-derived myelin, or secreted factors that protect host tissue. The relay test builds on reversible graft silencing (Dell’Anno et al., 2018; Section 2.1); the myelin and secretome branches are proposed experiments, not completed studies. Symbols are schematic and do not show measured anatomy; dashed arrows indicate predicted outcomes if a mechanism is required. Each experiment needs a selective intervention, confirmation that it changed the intended target, a viable graft, and appropriate controls. Rescue experiments can strengthen causal interpretation. Mechanisms may coexist, so partial effects are possible. A null result is difficult to interpret if the intervention fails. Outcomes should include motor, sensory, pain, autonomic, and daily function, as well as durability and safety.

**FIGURE 3:**
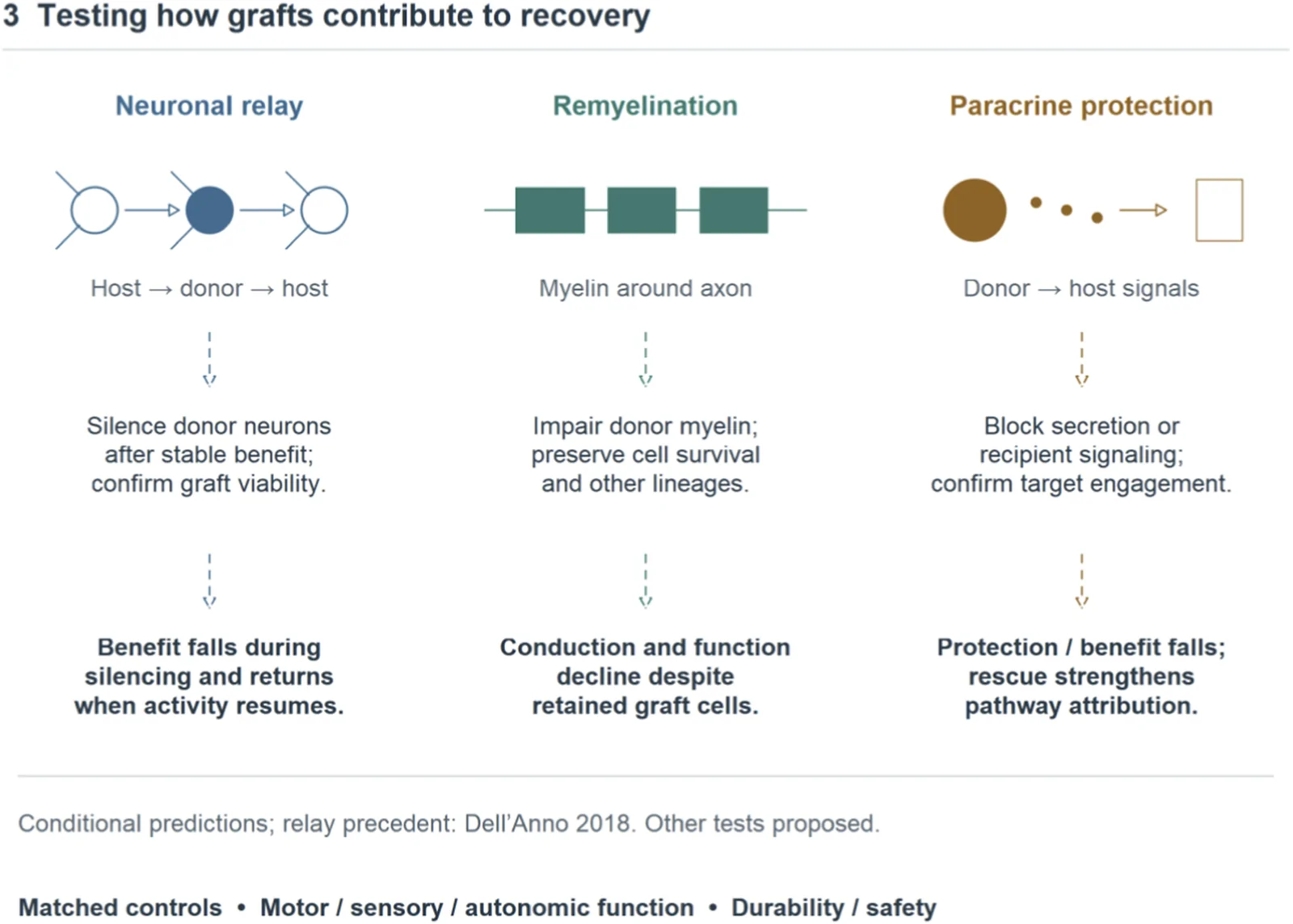
Experiments that discriminate routes to functional benefit.

### 7.1 Established contributions and supported conclusions

Molecular profiling has clarified which cell types and developmental states are present in the starting product and early graft. Single-cell assays resolve mixtures in the product, while donor-specific RNA analyses separate graft responses from changes in host tissue. The transplantation studies reviewed in Sections 2 and 5 show that regional identity and developmental stage affect graft behavior and that the lesion can redirect donor-cell phenotype. These findings guide product comparisons but do not identify one preparation that is optimal for every injury.

Secretome studies link the composition of cell-conditioned media to early host-tissue responses. Injury atlases show which cell populations, regulatory states, and tissue regions researchers can examine around grafts. Transplantation studies directly support immune modulation, tissue preservation, and remyelination in selected models. Physiological recordings and graft-removal experiments also show that some neural grafts contribute to host function. Molecular profiling helps identify the cells and processes involved; it does not by itself establish functional benefit.

Table 2 shows that donor profiling, paired RNA–chromatin measurements, and spatial metabolite mapping have mostly been conducted separately. Combining these measurements with longitudinal graft tracking and functional testing could determine whether starting-product features predict lasting recovery.

### 7.2 Unresolved links and limits of generalization

We still do not know how much recovery comes from donor-cell replacement, preservation of host tissue, remyelination, or greater use of spared circuits. Tracking donor and host changes over time can narrow these possibilities. Selectively disrupting each candidate process is needed to determine whether it contributes to a defined outcome.

Blood and cerebrospinal fluid sample compartments outside the graft site and can change with infection, medication, or systemic injury. Before using a biomarker to select recipients or monitor a product, studies must show that it reflects lesion biology and predicts a functional outcome.

Studies also need to test whether findings generalize across immune contexts and follow-up periods. Immunodeficient xenografts do not reproduce the full host immune response. Human grafts also mature over long periods in primates, making follow-up duration biologically important (Rosenzweig et al., 2018).

An early study reported extensive neural graft growth, relay formation, and functional recovery (Lu et al., 2012). A later reassessment confirmed graft outgrowth but found no significant locomotor advantage; it also reported partitions within the graft and limited host axon ingrowth (Sharp et al., 2014). This discrepancy suggests that axon number alone may not predict recovery. Tissue continuity and access between host and graft are additional features to test.

One lineage-tracing study attributed tissue protection to progeny from resident stem cells (Sabelström et al., 2013). Another found only a small, injury-dependent contribution from ependymal cells (Ren et al., 2017). For transplantation studies, label graft cells directly; a cell cluster that resembles ependymal or progenitor cells does not prove that it came from the graft.

### 7.3 Future perspectives and priority study designs

First, relate the administered product to later donor-cell states, host responses, and functional outcomes. Record both time since injury and time since transplantation. Collect tissue at intervals that capture donor survival, maturation, and functional recruitment (Supplementary Figure S1). Because spatial profiling usually requires tissue collection, use matched animal groups at each time point. Repeat behavioral, imaging, and physiological measures when possible. Stable donor labels or lineage barcodes, together with absolute cell counts, can distinguish donor-cell differentiation from selective survival or recruitment of host cells.

Second, use spatial molecular data to select candidate donor programs or host signals, then test them with selective perturbations. Include controls, confirm that the intervention changed its intended target, and use rescue experiments when feasible. Combine molecular measurements with axon tracing, electrophysiology, and blinded functional tests to identify which repair process changed. A loss of benefit cannot be attributed to the target if the intervention killed the graft or damaged host tissue. Even without behavioral improvement, experiments can show whether failure occurred during graft survival, maturation, communication, or functional output.

Third, test whether candidate markers predict outcomes across independent cell lines, manufacturing batches, and recipients before using them in potency assays or clinical biomarkers. Predefine outcomes and analyses, and link each animal or donor to its molecular data. Follow-up should monitor for delayed cell growth and immune complications, and measure motor, sensory, autonomic, and daily function. Focus on reproducible links between product features and repair rather than collecting more molecular data without a specific question.

These priorities require traceable batches, recipient context, sampling times, and independent biological replication; many cells from one animal do not represent multiple transplantation outcomes (Squair et al., 2021).

This review used a targeted search, not exhaustive screening, and did not formally assess risk of bias. We did not retain the original search yield, so the update log and bibliography cannot show how many studies were screened or excluded. Few transplantation studies combined multiple molecular layers with long-term functional outcomes. For articles whose full text was unavailable, we assessed only the abstract. These limits mean that conclusions should be interpreted study by study. They also point to the need for systematic evidence mapping.

## 8 Conclusion

Cell-resolved methods have clarified which cell types are present in a preparation, how graft cells change after transplantation, and which host cells and tissue regions respond to injury. Transplantation and functional studies show that, in selected models, grafts can protect tissue, add myelin to surviving axons, or communicate with host circuits. The next step is to measure the cell product, donor cells, host tissue, and function in the same study over time. Researchers should test whether candidate molecular features predict lasting benefit or directly contribute to it. Longitudinal sampling, selective perturbation, and independent replication should take priority over adding molecular measurements without a defined repair question.
