# Supplementary material for: Multi-omics insights into stem cell-mediated regeneration after traumatic spinal cord injury: cell fate, niche remodeling, and functional integration
Source: Front Cell Dev Biol. 2026 Oct 29;14:1994054. doi: 10.3389/fcell.2026.1994054 (PMC13755933; doi:10.3389/fcell.2026.1994054)
Supplement: Supplementary file 1 [file DataSheet1.pdf]

Supplementary material

Supplementary Note S1 Literature update and selection

This note documents the focused revision update completed on September 28, 2026. It is a narrative-review update, not a systematic review, and does not reconstruct an unavailable original screening denominator.

Sources and scope. Targeted web searches, including PubMed and publisher-domain searches, were followed by checks of PubMed/Europe PMC records, publisher pages, and Crossref metadata. Search concepts combined spinal cord injury with stem cell, neural progenitor, neural transplantation, single-cell, spatial, multi-omics, transcriptomic, epigenomic, proteomic, metabolomic, or machine-learning terms. Late-2025 and 2026 reports were specifically rechecked; there was no lower date limit for landmark work.

Selection. Published primary transplantation evidence was prioritized when it addressed product properties, donor fate, host responses, or function. Endogenous injury and external methods papers were retained for a stated graft-related question. Landmark selection reflected mechanistic or methodological contribution, not favorable results. Reviews supplied context, and published corrections were retained. Protocols and unpublished studies were not used as clinical outcome evidence.

Accounting. Eight recent research reports or records received targeted relevance assessment in this update: four primary reports were added, two already cited reports were retained, and two adjacent records were not added. Two guidance sources were added separately. The bibliography therefore increased from 73 to 79 records. These numbers describe this focused update only; 79 is not a count of independent experiments, trials, or screened studies. The original search yield and exclusion count were not recorded and are not estimated here.

Records assessed in the focused update

Added — Tucker et al. (2025), doi: 10.1038/s41467-025-65395-7. Direct neural transplantation evidence; physiological motor-circuit engagement without locomotor improvement. Publisher full text consulted.

Added — Zholudeva et al. (2026), doi: 10.1126/scitranslmed.aea7461. Human spinal interneuron grafting with RNA profiling, tracing, and electrophysiology. Interpretation limited to the PubMed abstract; no additional quantitative effect size is inferred.

Added — Sinopoulou et al. (2025), doi: 10.1038/s41587-025-02865-9. Primate transplantation, graft survival, and forelimb function. PubMed and accessible full-text sections consulted; survival-group comparisons are not described as randomized treatment effects.

Added — Abraham et al. (2026), doi: 10.1016/j.stemcr.2026.102926. Three-person chronic cervical cohort with 60-month follow-up. PubMed abstract consulted; distinct from the previously cited thoracic cohort.

Retained — Zhu et al. (2025), doi: 10.1038/s41551-025-01549-8, and van Niekerk et al. (2025), doi: 10.1038/s41586-025-09647-y. Already cited in Section 6.2; no duplicate bibliography entries added.

Not added as outcome evidence — Safety and early efficacy of iPSC-derived motor neuron progenitor cells in subacute spinal cord injury: protocol for a phase I, multicentre, open-label, single-arm trial (2026), PMID: 42419896. A protocol does not report observed efficacy.

Not added to the transplantation synthesis — Injury and therapy in a human spinal cord organoid (2026), doi: 10.1038/s41551-025-01606-2. An adjacent in vitro injury/therapy model rather than direct transplantation evidence; omitted to retain the graft-focused scope.

Guidance added separately — U.S. Food and Drug Administration, Potency Tests for Cellular and Gene Therapy Products (2011), and International Society for Stem Cell Research, Clinical Translation of Stem Cell-based Interventions (2025). These support product-development discussion and are not counted as research studies.

Supplementary Figure S1

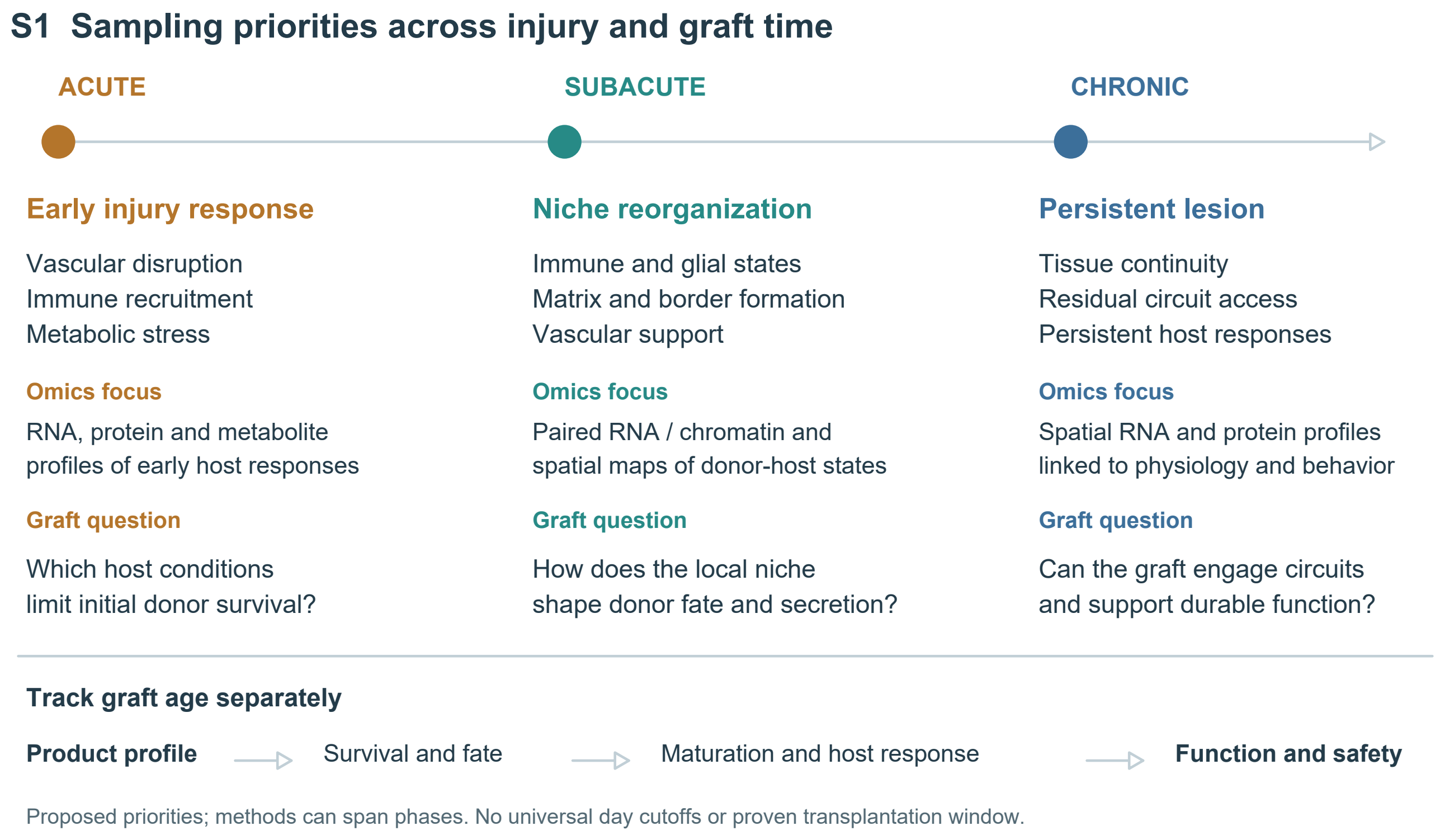

Sampling priorities across injury and graft time. The upper pathway organizes selected host features and graft-related questions across acute, subacute, and chronic SCI. These are biological phases with model-dependent boundaries, not fixed day cutoffs or a validated transplantation window. A separate graft clock begins with product characterization and implantation and follows survival, fate, maturation, host responses, function, and safety. A newly implanted graft can be present in a chronically injured host. Spatial tissue profiling generally requires parallel terminal cohorts, whereas selected imaging, physiological, and behavioral outcomes can be measured repeatedly. The diagram proposes sampling priorities synthesized from Sections 3, 6.2, and 7.3; it does not imply that all links have been validated within one study.
